# Policies to clean up toxic industrial contaminated sites of Gela and Priolo: a cost-benefit analysis

**DOI:** 10.1186/1476-069X-10-68

**Published:** 2011-07-28

**Authors:** Carla Guerriero, Fabrizio Bianchi, John Cairns, Liliana Cori

**Affiliations:** 1 London School of Hygiene and Tropical Medicine, Department of Health Research Services, London, UK; 2 Unit of Environmental epidemiology, CNR Institute of Clinical Physiology, Pisa, Italy; 3 Unit of Environmental epidemiology, CNR Institute of Clinical Physiology, Rome, Italy

## Abstract

**Background:**

Cost-benefit analysis is a transparent tool to inform policy makers about the potential effect of regulatory interventions, nevertheless its use to evaluate clean-up interventions in polluted industrial sites is limited. The two industrial areas of Gela and Priolo in Italy were declared "at high risk of environmental crisis" in 1990. Since then little has been done to clean the polluted sites and reduce the health outcomes attributable to pollution exposure. This study, aims to quantify the monetary benefits resulting from clean-up interventions in the contaminated sites of Gela and Priolo.

**Methods:**

A damage function approach was used to estimate the number of health outcomes attributable to industrial pollution exposure. Extensive one way analyses and probabilistic analyses were conducted to investigate the sensitivity of results to different model assumptions.

**Results:**

It has been estimated that, on average, 47 cases of premature death, 281 cases of cancer and 2,702 cases of non-cancer hospital admission could be avoided each year by removing environmental exposure in these two areas. Assuming a 20 year cessation lag and a 4% discount rate we calculate that the potential monetary benefit of removing industrial pollution is €3,592 million in Priolo and €6,639 million in Gela.

**Conclusions:**

Given the annual number of health outcomes attributable to pollution exposure the effective clean-up of Gela and Priolo should be prioritised. This study suggests that clean-up policies costing up to €6,639 million in Gela and €3,592 million in Priolo would be cost beneficial. These two amounts are notably higher than the funds allocated thus far to clean up the two sites, €127.4 million in Gela and €774.5 million in Priolo, implying that further economic investments - even considerable ones - could still prove cost beneficial.

## Background

It is estimated that approximately one-quarter of the global disease burden, and more than one-third of the burden among children, is due to modifiable environmental factors [1-3].

Materials, once widely used in industrial activities for their physical qualities, have proved carcinogenic, mutagenic and/or teratogenic for human health [4-8].

Priolo and Gela, in south-east Sicily, provide extensively documented cases of toxic contaminated sites where, due to the presence of large petrochemical industrial plants and to widely diffuse environmental pollution, several negative health effects have been observed. High levels of many chemical compounds have been detected in soil, water, groundwater, air sediments, fish and shellfish of both areas [9-13].

One recent descriptive study conducted in Gela and Priolo by the *Dipartimento Osservatorio Epidemiologico (DOE) *of the Sicilian Region showed excesses of overall mortality, of all cancer mortality and of many cancer and non-cancer cases when compared with regional and local reference levels [9]. These factors cause increasing concern in the local communities [14]. Nevertheless little has been done to reduce the exposure of the local population to pollution.

Cost-benefit analysis provides a common metric for evaluating costs and benefits arising from a given health policy and enables policy makers to pursue evidence-based strategies, to allocate resources efficiently and to prioritise the most beneficial interventions [15]. Importantly, the aim of cost-benefit analysis is not to assign a price to environmental-related health outcomes (e.g. cost of deaths) that have already occurred but to estimate, in monetary terms, the net benefits for society of averting future pollution-related health effects [16].

To date, there is a paucity of studies evaluating the net benefit arising from the reduction of environmental health hazards. The majority of published cost-benefit analyses focus on quantifying the monetary health benefit, and in particular the reduction in respiratory ailments, through air pollution control policies [17,18]. A few evaluations have considered other environmental-related health outcomes, such as cancer and hospital admission, but there have been no studies evaluating the potential net monetary benefits of reducing industrial pollution exposure [18-21].

This study, aims to quantify the long-term benefits resulting from the remediation of two highly polluted industrial sites: Gela and Priolo.

## Methods

**Figure 1:**
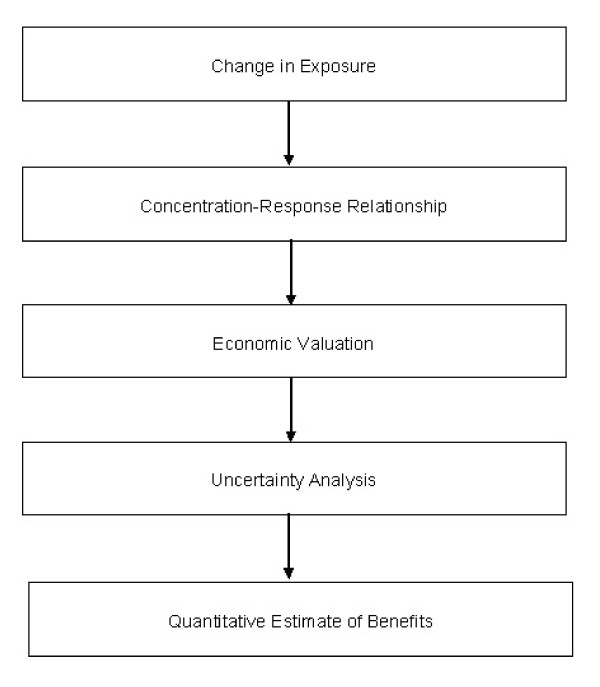
**Damage Function Approach**.

The analytical framework used to estimate the monetary benefit arising from the remediation of these industrial sites is the Damage Function Approach described in Figure 1[22].

This analytical framework combines both epidemiological and economic data to quantify how changes in exposure to environmental hazards affect the welfare of society. The first part of the study describes the environmental hazards present in the industrial sites of Gela and Priolo. In the second part, the health conditions attributable to environmental exposure are described, and quantified using recent epidemiological data. In the third part, monetary values are assigned to these health outcomes in order to estimate the monetary benefit arising from a comprehensive pollution control intervention in these industrial sites. The final part consists of performing extensive sensitivity analysis to account for parameter uncertainty.

### Health hazards arising from industrial activity in Gela and Priolo

The environmental pollution data in Gela and Priolo were collected in the context of the Reclamation Sites of National Interest policy. The pollution detected in Priolo involves soils and water and results from the release of ammonia, fluorhydric acid, chlorine, sulphur hydrogen, mercury; from discharges of industrial waste, inside and outside the site. Air pollution is caused by emissions of sulphur dioxide, nitric oxide, carbon dioxide, volatile organic compounds in the air (VOC). Groundwater is subject to depletion, withdrawal, and salinisation. Hydrocarbons, organic compounds and heavy metals pollution have been detected. The analysis of pine-needles has detected the presence of heavy metals [11]. There is also evidence of ecological systems disturbance and food chain contamination [11-13].

The groundwater inside the Gela remediation site contains arsenic, benzene, 1,2 dicloroethane, vinyl chloride, and mercury greatly in excess of legal limits [11]. Only temperature, turbidness, colibacteria, and iron presence have been analysed in drinkable water, and no data are available for other chemical compounds [11]. There are several abnormal data: toxic compounds in the air (benzene, non-metanic hydrocarbons, ozone, PM 10, VOC). Again the analysis of pine-needles has detected the presence of heavy metals[11]. Marine sediments have been found to be polluted by copper, arsenic, mercury and Polychlorinated biphenyls, showing toxicity in ecotoxicological analysis; fish and benthic organisms are polluted by heavy metals. The two rivers in the Gela plain are polluted by pesticides, copper, and zinc [11-13].

### Health outcomes attributable to exposure to industrial pollutants

Since the early 1980s, several epidemiological studies have been conducted in Sicily to investigate the health status of the populations living near industrial sites [23-25]. The most recent epidemiological study conducted by the DOE collected mortality data (from 1995 to 2002) and hospital discharges (from 2001 to 2006), for residents in the municipalities included in the high risk areas [9]. The health outcomes considered were: mortality from all causes, mortality by specific causes (e.g. infectious disease), hospital admission for all causes and disease specific hospital admission (e.g. hospital admission for lung cancer). Standardised mortality/hospitalisation ratios (SMR, SHR) were calculated by dividing the observed cases (e.g. individuals with lung cancer) by the expected cases. Estimates were reported for males and females separately and adjusted by age and socioeconomic deprivation [9].

The potential health benefits arising from a reduction in exposure to industrial pollutants are quantified for both Gela and Priolo, by considering the impact on total mortality, hospital admissions for cancer and non-cancer causes. For each of the selected health endpoints the population disease proportion attributable to the environment - the number of health cases that would not have occurred in the absence of the risk factor - was estimated using the following formula:

Where: a is the health outcome, b is gender, SMR/SHR is the Standardised Health Ratio obtained from the epidemiological study and n is the number of years over which epidemiological data have been collected. Upper and lower values for each estimate are calculated using the 95% CI of the SHR.

### Economic evaluation

#### Monetary valuation of environmental health benefits: methodologies and issues

Two approaches are used to assign a monetary value to the adverse health effects in environmental cost-benefit assessment. The cost of illness approach usually considers the direct medical cost, third party costs and productivity losses [26]. The second and more common approach is the willingness to pay (WTP) approach, which measures how much individuals are willing to pay for a reduction in the risk of a given adverse event (e.g. reduction in the mortality risk)[26]. Unlike the cost of illness approach, WTP includes the evaluation of intangible costs associated with adverse health events, for example, pain and fear, and account for individual preferences [26].

Several issues need to be considered when assigning monetary values to environmental health benefits. Contextual factors, such as individual characteristics, nature of the health outcome and number of individuals affected by the clean-up policy (e.g. death for cancer) have been found to be important determinants of WTP [27-29]. Finally, another important issue to consider in the evaluation of the benefits of a clean-up policy is the time lag (also referred to as the "cessation lag") between the clean-up policy and the onset of its related benefits.

#### Value of future reductions in mortality risk

An extensive literature search was conducted to find studies evaluating the value of a statistical life (VSL) for individuals exposed to environmental hazards. The Italian Government have not recommended values to use in Cost Benefit analysis of environmental health interventions. The baseline and the upper values, €5,800,000 and €6,300,000, selected for the analysis have been taken from a study conducted in four Italian cities with significant problems related to contaminated sites. Both values reported have been inflated to 2009 prices using the Harmonised Index of Consumer Prices [27]. The Alberini et al. study presents several novel elements. The study took into account for the first time how the WTP for mortality risk reduction is affected by the permanence of clean-up intervention and the size of population affected by the intervention [27]. The upper value used in this study was estimated assuming that the population living in the area covered by the program is 1 million, while the intermediate estimate is the baseline estimate suggested by Alberini et al. [27]. The lower estimate used, €2,100,000 is the European Commission's value of a statistical life (VSL) for environmental cost benefit analysis inflated to 2009 prices [30]. It is based upon a number of Contingent Valuation studies. Although it adjusts for the age of victims of environmental hazards it probably underestimates the VSL because it was estimated in the context of transport fatalities and does not consider fatalities specifically as a consequence of environmental hazards [30].

#### Value of reductions in risk of future negative health outcomes

Individual WTP might vary according to the cause of the hospital admission (e.g. cardiac versus respiratory hospital admission). As Pearce [31] suggests, the WTP to avoid cancer is higher than with other types of diseases because of the dread and pain effects associated with this pathology. For this reason, in the present study the two health end-points, cases of cancer and non cancer hospital admissions, are considered separately [31]. Estimates of the value of a statistical case of cancer were retrieved from a conjoint choice analysis conducted among 400 individuals who live in the industrial complex and contaminated site of Marghera (Venice) [28]. The baseline estimate of €2,656,000 is used in the present analysis. The upper estimate of €5,312,000 is the value of a case of cancer for high income individuals (annual household income more than €32,000). The lower estimate, €1,647,000 is for those individuals living farthest (more than 2.5 Km) from the contaminated sites [28].

Unfortunately, no studies have been conducted to estimate the WTP to reduce the risk of a hospital admission in the context of exposure to toxic pollutants. The estimated WTP to avoid a hospital admission comes from the ExternE Project (€9,500 inflated to 2009 prices) [31]. Although this value is heavily dependent on US studies, it is the only WTP estimate of the monetary benefit of averting a hospital admission [31,32].

#### Cost of reclaiming Gela and Priolo

It is difficult to establish whether a remediation strategy will attain the forecasted health improvements within the planned budget. The costs of long term remediation projects accumulate over years and effectiveness is only observable after a long time [33]. Further, sometimes it is not possible to identify all the sources of environmental externalities to be addressed by the intervention (e.g. toxic waste from illegal dumping is often not visible). Frequently the estimated cost of a regulatory intervention is only the abatement expenditure (e.g. the cost per ton of emission reduction) which, as Kopp et al. [34] suggest is a narrow measure of the cost of regulatory compliance.

In the case of the Italian Reclamation Sites of National Interest, a budget is established for each intervention plan and approved by the Ministry of the Environment. The polluter is supposed to implement and pay for the clean-up. If the polluter is not identified or is not acting to reclaim the site, the closure of the process and cost negotiation is established by means of a Memorandum of Understanding. This is the case for Gela and Priolo where the final cost of clean-up remains uncertain. To date the agreed document identifies €774.5 million for Priolo and € 127.4 million for Gela. However these estimates cover only some of the interventions required [13,35-37].

The present value of the potential monetary benefits (PVB) arising from the clean-up of the sites was estimated using the following formula:

Where: λ is the WTP to avert the health outcome (e.g. WTP to avert a case of premature mortality),Xa is the number of health endpoints averted by the clean-up of Gela and Priolo industrial plants (e.g. number of non-fatal cancers averted), t is the number of years over which the benefits accrue, and d is the discount rate [19].

There is uncertainty regarding the length of time over which a clean-up intervention will display its benefit. The permanence of clean-up depends on two elements: the intrinsic composition of the contaminated site and the type of remediation technology adopted. For example, if the cheapest technology is implemented, e.g. capping the contaminated site, the benefit will last for the shelf-life of the cap [38]. Another important element to consider is how long it will take to observe a decline in the number health outcomes associated with contaminated sites (also referred as cessation lag). The duration of the cessation lag is likely to vary by type of health outcome (shorter for mild adverse events such as asthma and bronchitis, and longer for more severe events such as cancer). A recent US study assumes that four years after clean-up it is possible to observe a 20-25% decline in the number of congenital anomalies [39]. There is currently uncertainty regarding the type of clean-up technology to adopt in both sites. In order to facilitate comparison with previous studies the period of time over which policy benefits arise and the policy latency in the baseline scenario are assumed to be 50 and 20 years respectively [19]. Extensive one way sensitivity analyses have been performed to evaluate the robustness of the results to these model assumptions.

Discounting plays a crucial role in determining the future monetary benefits/costs of environmental health interventions, especially for long-lasting health benefits (e.g.100 years). While there is a broad consensus that future costs and benefits should be discounted, there is little agreement on both the discounting model and the discount rate to use [40].

According to the discounted utility model (also known as the constant rate or exponential model) individuals' intertemporal preferences are time consistent [41,42]. Although this model is generally used in economic evaluation, studies of animal and human behaviours generally show that individual preferences are dynamically inconsistent. Individuals tend to display higher discount rates over short time horizons and lower discount rates over long time horizons [39,41-45]. Such a relationship conflicts with the discount utility model's assumption of a constant discount rate, and is a characteristic of hyperbolic models. Despite the potential descriptive relevance of hyperbolic models they have rarely been used normatively in the evaluation of the consequences of environmental decisions and the discounted utility model remains the standard [40].

According to Gravelle and Smith [46], the majority of the studies use a discount rate ranging between 3 and 5%, and usually extensive one way sensitivity analyses are performed to assess results robustness to variation of discount rate. In the present study the future health benefits are discounted using a constant 4% discount rate as recommended by the European Commission [30].

The final step of cost benefit analysis is the selection of the decision rule to evaluate whether the intervention is worth-while [47]. The information on costs and benefits is combined in a single indicator the Net present Benefit (NPB):

Where PVB is the present value of the health benefits (averted deaths, hospital admission for cancer and non-cancer causes) and PVC is the present value of the cost of cleaning up industrial pollution in Priolo and Gela. lean-upIf the NPB is positive the intervention is cost beneficial otherwise the clean-up option is not deemed socially worthwhile. NPB values can be also use to rank the clean-up of different sites within for example the Superfund budget and to prioritize those sites with higher NPB values [47].

### Sensitivity analysis

#### One-way sensitivity analyses

Environmental health benefits arising from a pollution control policy are not marketable goods and as a consequence their value is highly uncertain. Univariate deterministic analyses were performed in order to estimate the impact of uncertainty on the results.

To estimate the impact of the discount rate and to facilitate the comparison of the study findings with other European studies, analyses are presented using a 7% discount rate, as estimated by Alberini et al. [27] and 2% and 4% as recommended for cost-benefit analyses by the EC [30].

It is unknown whether or not the clean-up interventions that have been planned in Gela and Priolo will produce a permanent or a temporary reduction in pollutant exposure. According to Alberini et al. [27] different types of remediation policies would lead to different degrees of permanence of the health risk reduction. The number of years over which the risk reductions would be observed would be higher for permanent remediation compared to temporary remediation (contaminant containment interventions).

It is also uncertain for how long the emissions of toxic compounds will last if clean-up and stricter controls are not undertaken. Improvements in the technology adopted by the factories (for example the introduction of the SNOx chimney stack in the Gela ENI factory) and the closure of several highly polluting industrial plants (e.g. the closure of the Eternit factory that used to produce asbestos and cement) suggest a decline in the emissions in the two areas. The presence of landfills (e.g. a 8 million m2 landfill of phosphogypsum) and the lack of ordinary maintenance (e.g. several leakages of oil from refinery holding tanks have been discovered) are the cause of current emissions and they are likely to continue for a long time. Environmental data collected in Gela and Priolo reveal that the concentrations of several harmful substances exceeded the limits established by the law. For instance, in the groundwater of Gela arsenic concentrations reached the value of 250.000 μg/L versus the law established limit of 10 μg/L [11]. A study conducted in Michigan found that the ingestion of arsenic can be fatal even at very low concentrations[48], In the absence of further releases of this toxic substance, its concentration in the Gela groundwater will decrease with time. But, it is uncertain how long it will take to reach levels safe for human health.In order to explore differences in time to failure of the remedies a one way sensitivity analysis was performed assuming three time frames: 10 years (for a contaminant containment intervention), 50 years and 100 years (for permanent remediation policies with long lasting benefits).

#### Probabilistic sensitivity analysis

To further explore uncertainty a probabilistic sensitivity analysis was performed. Probability distributions were assigned to important components of the analysis [34]. WTP estimates and cessation lags were sampled from a gamma distribution while normal distributions were adopted for the number of excess cases [49]. Using a Monte Carlo simulation 10,000 samples were generated from parameter probability distributions [50]. The costs, the benefits and the expected net benefit were calculated for each simulation according to the following formula:

The results of the simulations were presented as a Cost-Benefit Acceptability curve (CBAC) using standard methodologies [51]. A CBAC shows the probability that a reclaim policy is cost beneficial for a range of clean-up intervention costs by plotting the proportion of simulations for which the net benefit of the remediation policy is positive for reclamation costs ranging from €127.4 to €12,000 million in Gela and from €774.5 to €4,000 million in Priolo. The lower bounds are the sums agreed to date for the clean-up of the sites. The higher bounds are the cost at which the clean-up has a zero probability of being cost beneficial. The opportunity cost and the effectiveness of a clean-up policy on pollution-related health outcomes are difficult to estimate a priori. In the case of Gela and Priolo remediation is still at an early stage and epidemiological evidence on the effectiveness of clean-up interventions is not yet available. In order to account for the uncertainty around the cost and the effectiveness of remedial interventions in the two areas four CBACs were constructed assuming different levels of remedial effectiveness (20%, 50%, 80% and 100% of the health outcomes will be averted). The lower is the effectiveness (percentage of health outcomes averted) the lower is the probability that the remedial intervention is cost beneficial. When more specific epidemiological evidence is available CBACs will allow policy makers both to gauge the cost effectiveness of interventions and to improve environmental site remediation through performance based environmental management.

## Results

The health outcomes attributable annually to industrial pollution exposure in Priolo and Gela were estimated using data from Cernigliaro et al. [9]. As shown in Table 1, a reduction in exposure to environmental pollution in Priolo would avert 8 (2-11) premature deaths, 118 (85-151) -cancer related hospital admission and 692 (587-780) non cancer hospital admissions each year; while in Gela would avert 39 (12-64) premature deaths, 163(134-192) cancer and 2,010 (1,912-2,095) non cancer hospital admissions each year.

**Table 1:** Annual health outcomes attributable to pollution exposure in Gela and Augusta-Priolo areas

	Gela		Priolo	
	**SHR(95%CI)**^**a**^	Annual Cases	**SHR(95%CI)**^**a**^	Annual Cases
**Mortality**				
Male	106 (102-109)	23 (8-35)	110 (102-118)	8 (2-11)
Female	105 (101-109)	16 (4-29)	NS	NS
**Cancer hospital admissions**				
Male	115 (110,5-119,7)	53 (38-67)	116 (111.6-119.8)	69 (53-85)
Female	127 (122,8-131,9)	110 (96-125)	110 (106.3-114)	49 (32-66)
**Non cancer hospital admissions**^**b**^				
Male	121 (119-122)	909 (864-952)	107 (105.7-107.7)	413 (360-482)
Female	124 (122-125)	1,101 (1,048-1,143)	104 (103.5-105.4)	279 (227-298)

a SHR: Standard Health Ratio; b Number of hospital admission for all causes minus cancer-related hospital admissions

Assuming a 20 year cessation lag, a 4% discount rate and that the benefits will last 50 years the potential monetary benefit from abating industrial pollution in Gela and Priolo was estimated for each health outcome separately (Table 2).

**Table 2:** Monetary Benefits (Million€,2009 values) of site remediation

Item	Gela	Priolo
**All death**	2,203 (247-3,933)	455 (41-676)
**Cancer hospital admissions**	4,248 (1,918-10,000)	3,072 (1,372-7,864)
**Non cancer hospital admissions**	149 (149-160)	53 (47-76)
**Total benefit**	6,639 (2,314-14,093)	3,592 (3,167-3,802)

As expected, due to the many health outcomes each year associated with exposure to pollution the potential monetary benefit of site remediation in Gela and Priolo is high. In Gela it ranges between €2,314 million (the low SHR and low WTP scenario) and €14,093 million (the high SHR and high WTP scenario), with €6,601 as baseline value. In Priolo, where the health outcomes, and in particular the number of premature avoidable deaths are lower, the potential monetary benefits of site remediation would be €3,592 million (3,167-3,802).

Given the predicted cost of clean-up policies in the two areas, €774.5 million in Priolo and €127.4 million in Gela, the potential net monetary benefits of reducing industrial pollution exposure were estimated to be €2,817 and €6,521 million respectively. This implies that if the pollution control policies that have already been identified are not effective in reducing the impact of pollution exposure on health, it would be worth spending up to €6,521 million in Gela and €2,871 million in Priolo on a completely effective reclamation.

### One-way sensitivity analysis

Extensive one way sensitivity analyses were performed to assess the robustness of study findings to parameter uncertainty.

In Table 3 the net benefit of pollution control policies are reported assuming different time horizons for the benefits and different discount rates. Given an estimated cost of €127.4 million of reclaiming the area, the potential benefits are always higher than the cost in Gela, while in Priolo when benefits are discounted at a 7% discount rate, as suggested by Alberini et al. [27] the pollution control interventions are not cost effective if the benefits arising from the remediation only last 10 years (Table 2).

**Table 3:** Net benefits (million €,2009 values) by time horizon over which the benefits accrue each year.

	100 year time	50 years time	10 year time
**Gela**			
**7% discount factor**	2,364 (1,332-3,305)	2,287 (1,285-3,193)	1,094 (591-1,562)
**4% discount factor**	7,403 (2,512-15,936)	6,474 (2,187-13,965)	2,365 (1,340-3,306)
**2% discount factor Priolo**	13,116 (7,667-18,187)	9,529 (5,556-13,226)	2,632 (1,497-3,689)
**7% discount factor**	576 (417-656)	528 (378-608)	-99 (-170;-53)
**4% discount factor**	3,419 (2,948-3,672)	2,806 (2,393-3,027)	613 (458-697)
**2% discount factor**	6,602 (4,091-8,253)	4,464 (2,592-6,077)	722 (239-1,107)

### Probabilistic sensitivity analysis

**Figure 2:**
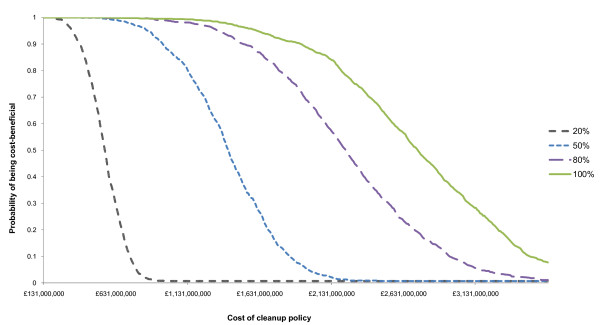
**Cost Benefit Acceptability Curves of Priolo clean-up assuming different remedial effectiveness**.

**Figure 3:**
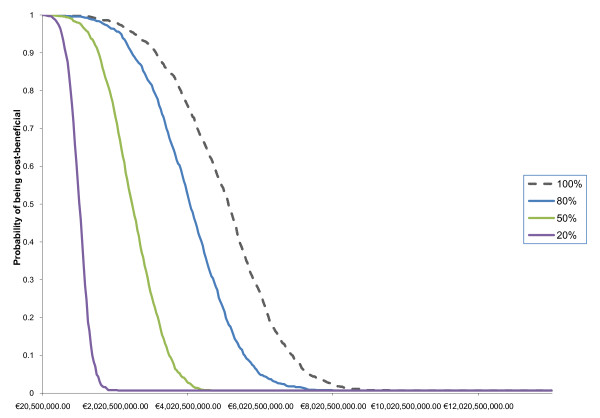
**Cost Benefit Acceptability Curves of Gela clean-up assuming different remedial effectiveness**.

Figures 2 and 3 report the probabilistic sensitivity analyses for Gela and Priolo respectively. The interpretation of the CBACs is straightforward. The lower the efficacy of clean-up policies the lower is the probability of being cost beneficial. For example, in Priolo a remedial intervention with low effectiveness (preventing only 20% of health outcomes) is unlikely to be cost effective if it costs more than €700 million.

As expected in Gela, pollution control policies are more likely to be cost beneficial even for high clean-up costs. In this area, assuming that 100% of the health outcome attributable to pollution will be averted a pollution control policy costing €7,000 million has 50% probability of being cost beneficial. In Priolo, on the other hand, a pollution control policy costing more than €3,000 million is unlikely to be cost beneficial even if all the negative health outcomes attributable to industrial pollution exposure were to be averted.

## Discussion

Assuming the excesses of standardized mortality or hospitalization ratios were attributable to environmental pressures documented in the areas, avoidable cases were estimated using regional health statistics [9,10]. Although these data are currently collected and controlled through standardized methods for epidemiological and public health purposes some limitations should be considered the existing studies design does not allow to assessing the causal relationship between industrial pollution exposure and health. However it should be noted that the proportions of deaths and non-fatal cancers attributed to the environment are comparable to those suggested by WHO and other authors [1,3]

Using epidemiological evidence from the DOE study this economic evaluation quantified the number of health outcomes attributable to industrial pollution exposure in the two areas of Priolo and Gela[9].

The present study suggests that, 47 premature deaths, 281 cancer related hospital admissions and 2,702 non-cancer hospital admissions could be avoided each year by removing the environmental exposure of the communities in these two areas.

Given the potential health benefits, the estimated monetary gain of an effective pollution control policy would be €3,592 million in Priolo and €6,639 million in Gela. The cost of removing contamination from the two sites is uncertain. To date, the cost of the clean-up interventions planned by the Ministry of Environment [35-37] are €774.5 million and €127.4 million for Priolo and Gela respectively. If these were the true costs of clean-up, then the net monetary benefits arising from clean-up would be extremely high. If on the other hand, further investments are necessary to avert pollution related health outcomes, this study suggests that any further intervention costing less than €2,817 million in Priolo, and €6,521 million in Gela would be cost effective (the benefit outweighs the cost).

The study has strengths and limitations. This analysis used only WTP estimates based on CV studies to determine the potential benefits of averting morbidity and mortality arising from pollution control policies. WTP is preferred to cost of illness because it takes account of all the costs associated with a given health effect (e.g. suffering, loss) and thus provide a better estimate of the potential benefits [52].

A further strength of this study is that it allows for differences in WTP for different health effects. In order to account for the cancer premium, the benefits of averting non fatal cancers and hospital admissions were evaluated separately.

A further advantage of this study is that it uses probabilistic sensitivity analysis to address simultaneously uncertainty regarding the parameters of the model. For the first time, in the context of environmental cost benefit analysis, this work used cost benefit acceptability curves in order to capture the uncertainty around the estimated net benefit and to show the probability that intervention will be cost beneficial, given a range of clean-up policy costs and different degrees of effectiveness of remedial interventions.

Nevertheless, there are several limitations to the study. It was assumed that the excess mortality, cancer and non cancer hospitalization are attributable to the environmental pressures, that represent the main difference between the study areas and the reference areas (not only the whole Sicily region but also a limited number of neighbouring municipalities) [9]. The absence of studies with an analytical design that would provide better evidence of the causal relationship between environmental pressure and health is a limitation for the present analysis. Extensive deterministic and probabilistic sensitivity analyses were conducted to address this element of uncertainty.

For example, this study provides only a partial estimate of the overall benefit obtainable with the clean-up of the two contaminated sites in Gela and Priolo. Excess congenital malformations, mainly uro-genital anomalies and particularly hypospadias, in these areas suggest a plausible association with exposure to documented pollutants [53,54]. However, because there are no conclusive etiological studies, or studies estimating the WTP to avert cases of congenital malformation these potential benefits were not included.

Furthermore, the analysis excludes the potential benefits for the ecosystem related to increased agricultural and fishing productivity and also the increasing quality of environmental resources such as rivers and ground waters and the sea [52].

Finally, although results were presented separately, in terms of average, high and low estimates, it was not possible to adjust the WTP values for characteristics of the Gela and Priolo populations (e.g. income and education) and by the nature of the clean-up interventions (e.g. temporary versus permanent) [28,27].

In 1993, President Bill Clinton's executive order 12866 established that government and private parties should be fully informed about the costs and the benefits of regulatory options [55]. While a significant volume of work has evaluated the cost effectiveness of air pollution regulations; cost effectiveness analysis has rarely been used to prioritise contaminated sites and select clean-up interventions [55]. As long as the true benefits of clean-up interventions are unknown it will be impossible to allocate efficiently the limited funds available.

## Conclusions

In 1998, law 426/98 established Priolo and Gela among the first 15 Italian sites included in the National Reclaim Program [56]. Nevertheless, the damage caused to the environment and the impact on human health by industrial pollution has yet to be fully assessed sufficient to address the complete *continuum of public health*, from pollutants emission to human exposure to disease [57].

In this situation where "facts are uncertain, values in dispute, stakes high and decisions urgent" Funtowicz and Ravetz [58], it is very difficult to reduce the uncertainties, for example, regarding causal mechanisms in environmental health, consequently it is necessary to accept uncertainty and move forward. The present study proposes a methodology for the economic evaluation of the health effects of environmental pollution and can contribute a basis for the prioritisation of interventions.

This study suggests that clean-up policies costing up to €3,592 million in Priolo and €6,639 million in Gela would be cost beneficial. Given the cost of the planned clean-up interventions -€127.4 million in Gela and €774.5 million in Priolo these results suggest that if additional spending was required in order to eliminate the impacts on health, as long as the total expenditure required was less than €6,521 million in Gela and €2,871 million in Priolo, reclamation would continue to be a cost-effective investment.

## List of Abbreviations

DOE: Dipartimento Osservatorio Epidemiologico; VOC: Volatile organic compounds in the air; SMR: standard mortality ratio; SHR: standard health ratio; VSL: value of a statistical life; PVB: present value of benefit; PVC: present value of cost; CV: contingent valuation; CBAC: cost benefit acceptability curve.

## Competing interests

The authors declare that they have no competing interests.

## Authors' contributions

All the authors contributed to the study design, data collection, and interpretation of results and reviewing of the manuscript. CG led the design of the research, drafted the paper and conducted the analysis. JC co-lead the design of the economic model, data analysis and interpretation. FB and LC contributed substantially to the draft of the paper and provided critical inputs on the probabilistic analysis design and on the epidemiological and environmental data interpretation. All authors read and approved the final version of the manuscript.
